# Determinants of the persistence of malaria in Rwanda

**DOI:** 10.1186/s12936-020-3117-z

**Published:** 2020-01-21

**Authors:** Guillaume Rudasingwa, Sung-Il Cho

**Affiliations:** 1 Department of Public Health Sciences, Graduate School of Public Health, Seoul National University, Seoul, 08826 South Korea; 2 Department of Public Health Sciences, Graduate School of Public Health, Institute of Health and Environment, Seoul National University, Seoul, 08826 South Korea

**Keywords:** Malaria, Mosquito net, Season, Altitude, Residence, Wealth category

## Abstract

**Background:**

Malaria has a considerable impact on the health of the populations of developing countries; indeed, the entire population of Rwanda is at risk of contracting the disease. Although various interventions to control malaria have been implemented in Rwanda, the incidence of malaria has increased since 2012. There is an interest in understanding factors driving its persistence in Rwanda. This study aims at evaluating the effect of socio-economic and environmental factors, seasonality and the use of insecticide-treated mosquito nets (ITNs) on malaria persistence in Rwanda.

**Methods:**

This study analysed data from the 2014–2015 Rwanda Demographic and Health Survey of 11,202 household’s members composed of children under the age of 5 and women aged between 15 and 49. Bivariate analysis was performed between the outcome and each covariate including wealth, altitude, education level, place of residence, and use of ITNs generating percentages. Chi square test was performed to compare malaria negatives and positives on each covariate. Significant variables were subjected to logistic regression analysis to evaluate factors that are significantly associated with malaria at P < 0.05. The analysis was performed in R x64 3.6 and QGIS3.6 was used to map geographical distribution of malaria cases.

**Results:**

The lowest wealth category was associated with the incidence of malaria [AOR] = 1.54, 95% CI (1.78–2.03). Having a place of residence < 1700 m above sea level (asl) and non-use of ITNs were significantly associated with the incidence of malaria (adjusted odds ratio [AOR] = 2.93, 95% confidence interval [95% CI] 1.94–4.42 and [AOR] = 1.29, 95% C.I (1.03–1.60), respectively). Season and type of residence were not significantly associated with malaria prevalence while women had lower risk of contracting malaria than children.

**Conclusion:**

Increased malaria prevalence was associated with lower income, non-compliance with bed-net usage and living below 1700 m of altitude. In addition to current malaria control strategies, potential interventions in individuals with lower income and areas at low altitudes should be taken into consideration when formulating malaria-control strategies, Also use of ITNs to control the spread of malaria should be emphasized.

## Background

Malaria is a major threat to human health globally and is endemic to the tropics and subtropics. Because of its impact on public health, malaria has received much attention [1]. Rwanda is a sub-Saharan African nation whose entire population is at risk of contracting malaria. In Rwanda, 1.8 million and 443,000 children < 5 years of age and pregnant women, respectively, developed the disease in 2016 [2]. Of the 30 districts of Rwanda, 19 are prone to epidemics and malaria is endemic in 11. The western and northern regions of Rwanda (~ 63% of the country) are epidemic-prone, while other areas are categorized as endemic and stable malaria-transmission zones, with the major foci in the south-eastern and eastern regions [2].

The economic status of affected populations is a challenge for malaria control programs [3]. Poverty is closely associated with malaria, and the risk of malaria is two-fold greater in the poorest compared to the richest children in a community [4]. Poverty is associated with the incidence of malaria in Uganda [5]. Therefore, information on the socioeconomic conditions of malaria-affected populations is needed to control the transmission. However, few studies have evaluated the socioeconomic factors related to the transmission of malaria in Rwanda.

The World Health Organization (WHO) recommends that all individuals at high risk of malaria should have access to and use long-lasting insecticidal nets (LLINs) [6] and various studies identified the effectiveness of ITNs in malaria reduction among children and pregnant women [7, 8]. Strategies for controlling malaria, such as insecticide-treated mosquito nets (ITNs), significantly reduced malaria transmission in Rwanda from 2006 to 2008 [9]. In Western Kenya, difficulty in using net among children under 5 years was related to common reasons like lack of where to hang Nets, its shape and distance between point of hanging and bedding level [10]. Although high coverage of ITNs has been achieved, a trend towards non-use of ITNs has been noted in the Eastern Province due to hot season, bed bug infestation and due to unknown reasons [11]. In contrast, little is known about the misuse or non-use of ITNs among the general population across the country.

Elevation above sea level or altitude is one of the important factors that determine malaria transmission patterns and the probability of transmission of malaria decreases with increasing altitude [12]. This is because the temperature at high altitudes is lower than that required for development of the malaria parasite and for the activity of insect vectors. Information on the effect of altitude on the transmission of malaria and the local pattern of malaria transmission is essential for planning interventions. However, little is known about the effects of altitude and environmental factors on malaria transmission in Rwanda.

This study evaluated the associations between the incidence of malaria and socioeconomic factors, altitude, place of residence, season, and non-use of ITNs. The results could possibly facilitate the formulation of effective strategies to reduce and ultimately eliminate malaria in Rwanda.

## Methods

### Study design

The RDHS 2014–2015 adhered a two-stage sample design intending to nationally estimate key indicators as well as rural and urban areas, five provinces (Kigali city, Southern, Western, Northern and Eastern) and each of Rwanda’s 30 districts. The first stage consisted of selecting clusters or sample points composed of EAs (Enumeration areas) delineated for the Rwanda Population and Housing Census 2012. A total of 492 clusters were selected, where 113 are located in urban areas and 379 in rural areas. The second stage consisted of household’s systematic sampling. From July 7 to September 6 in 2014, listing of households was undertaken in all selected EAs and households to be surveyed were randomly selected from these lists. From each sample point, 26 households were selected making a total sample size of 12,792 households. A representative sample of 11,202 household members composed of children under the age of 5 and women aged between 15 and 49 was analysed in this study.

### Rwanda Demographic and Health Survey 2014–2015

From October 5 2014 to November 2 2014, field workers have been trained by NISR (National Institute of Statistics in Rwanda) with support from ICF (Inner City Fund) International to conduct RDHS 2014–2015. The training was composed of class presentations by trainers, group practice, mock interviews, and role playing among participants in the classroom. Some brief presentations were made by experts and guest speakers from the Ministry of Health (MoH) of Rwanda, RBC (Rwanda Biomedical Center) and UNICEF (United Nations International Children’s Emergency Fund) about national health strategies related to malaria, maternal and child health, nutrition, contraception and HIV.

Field workers were trained about the questionnaires through October 26 2014 and 34 participants were identified as health technicians and were separated and trained on biomarkers from October 27 to 30. The remaining participants continued to be trained on the questionnaires. Representatives from the NRL with support from ICF International conducted trainings on biomarkers for fieldworkers. Health technicians received trainings about how to prepare blood slides for malaria testing, and how to conduct anemia and rapid malaria testing as well as procedures for handling and packaging dried blood spots and slides [13].

### Fieldwork

The totality 17 teams participated in data collection for the 2014–15 RDHS from November 9 2014, to April 8 2015. Vehicle and driver were provided for each team and all blood specimens and questionnaires were transferred to the office of NISR every 3–4 days. All the field activities were coordinated and supervised by supervisors from the NISR and NRL/RBC.

### Data processing

2014–2015 RDHS data processing began as soon as questionnaires were received from the field. The numbers of questionnaires and malaria slides were verified by receptionists. Questionnaires were then checked and open-ended questions were coded by trained editors. Malaria slides with transmittal sheets were sent to the Parasitological and Entomology Laboratory and tested for malaria. Using CSPro computer program, Questionnaire data were entered by trained data processing personnel coordinated by NISR data processing officer with technical assistance from ICF International during the entire data processing period [13]. The wealth index was created in three steps. Firstly, subsets of indicators which are common to both rural and urban areas were used to create wealth scores for households located in both areas. Secondary, for each household in rural and urban areas, using area-specific indicators, separate factor scores were produced and lastly the separate area-specific factor scores were combined to produce a nationally applicable wealth levels after adjusting for area-specific scores. Once the index were computed, national-level wealth quintiles ranging from lowest to highest were obtained by assigning scores to households members, ranking each person in the population by his or her score, and then dividing the ranking into five equal categories, each comprising 20 percent of the population [13]. Wealth was categorized as poorest, poorer, middle, richer, and richest. The highest education level was recorded as uneducated, primary and secondary or higher. Ownership of mosquito nets was recorded as yes or no, and sleeping under an ITN was recorded as yes or no. Malaria status was recorded as positive or negative. The district and type of residence were recorded as rural or urban. Month was recorded as January, February, March, April, October, November, or December, as malaria cross sectional surveys were conducted during these months.

### Malaria cross-sectional surveys

From November 9 2014 to April 8 2015, Rwanda Demographic Health and Survey were conducted by National Institute of Statistics of Rwanda (NISR) and Ministry of Health. Among the study population of 54,905 persons, a subsample of 50% of the households were selected and a totality composed of 11,202 women 15–49 years of age and children < 5 years of age who agreed to malaria testing across the country were subjected to malaria rapid test and blood smear diagnostic tests. Health technicians prepared blood slides for malaria testing and performed malaria rapid testing. Malaria testing were performed with non-reusable, sterile and self-retractable lancets in order to collect blood specimen from women and children under the age of 5. In the case of Rapid diagnostic tests, a drop of blood was obtained by pricking the end of the finger. Survey supervisors collected Blood samples for two or three times a week, then transmit samples to Rwanda National Institute of Statistics for verification then microscopic examination were done at the Entomology Laboratory. The results were then referred to the National Reference Laboratory/RBC (Rwanda Biomedical Center) for quality control and assurance. The results of malaria rapid tests and blood-smear tests were recorded as positive or negative [13].

### Seasonal pattern measurement

The RDHS 2014–2015 was conducted during January, February, March, April of 2015 and October, November and December of 2014; the amount of rainfall and the temperature varies among these months. The elevation above the sea level were classified as < 1700 and ≥ 1700 m. The prevalence of malaria was calculated in each of these months. The months were classified into rainy (March, April, November, and October) and dry (December, January, and February) seasons.

### Analytical principle and methods

#### Descriptive statistics

Using svyby command from survey library in R x64 3.4.4, descriptive statistics were performed with percentage and standard errors (SE) with 95% confidence interval to identify the distribution and use of mosquito nets by general characteristics, proportion of malaria cases per season and proportion of malaria at < 1700 and ≥ 1700 m asl and income category using RDHS sample weights, adjusting for cluster survey design. The significance between and each covariate malaria negatives and positives were analysed using Chi square test and significant variables were included in multiple logistic regression analysis.

#### Logistic regression analysis

Using svyglm command form Survey library, multiple logistic regression analysis was conducted to explore the factors affecting the prevalence of malaria. Logistic regression model was used to assess the association between prevalence of malaria and altitude, age, sex, season, use of mosquito nets, social economic factors (level of education, income level) and place of residence. The threshold for significance was set at *P* < 0.05, and results are presented as crude odds ratios (CORs) or adjusted odds ratios (AORs) and 95% CI. QGIS3.6 was used to map the prevalence of malaria detected using the rapid malaria test.

### Ethic statement

The Institutional Review Board of Seoul National University approved this study (IRB No. E1902/002-003). In addition, reporting of findings adhered to the STROBE guidelines for cross-sectional studies.

## Results

### Prevalence of malaria

Among the 11,202 subjects tested, 311 subjects’ results were missing remaining with 10,891 subjects. Among 10,891 subjects, 594 malaria cases were identified by the rapid malaria test, for a prevalence of 5.45%. The prevalence of malaria was high in the southern and Eastern Provinces. Kirehe District in the Eastern Province had the highest prevalence of malaria (19.69%), followed by Ngoma and Nyanza Districts in the Southern Province (14.47% and 12.57% respectively). No single case was found in Burera district in Northern Province.

### Prevalence of malaria according to altitude

Household members living at < 1700 m asl had 82.74% of all positive cases and only 12.26% for these living at ≥ 1700 m asl. According to malaria rapid tests results, Northern province which is the highly-elevated province in Rwanda showed to have lower malaria prevalence rate than others (Table 2).

### Mosquito net ownership and utilization

According to Table 1, 84.30% of the population own at least one mosquito net. However, only 66.25% of the population slept under an ITN. Province of residence, education level, and wealth category influenced the ownership of ITNs. The frequency of ownership of ITNs was highest in Kigali (93.58%) and lowest in the Western Province (72.14%). Highest income households were slightly more likely to own mosquito nets (94.09% and 68.93% among very high and very low wealth categories, respectively) (Table 1). Approximately 91.00% of the subjects with the highest education level owned mosquito nets, compared with 82.64% of the uneducated subjects. The use of ITNs was influenced by the same factors as their ownership. The percentage of the subjects who slept under an ITN on the night preceding the survey was highest in Kigali (78.67%, and lowest in the Western Province (55.44%). The proportion of urban residents who slept under an ITN the night before the survey was higher than that among rural residents (75.24% and 63.46% respectively). The rate of ITN use increased with increasing wealth category. The frequency of ITN use in the very low income category was 51.67% compared to 78.73% for the very high income category.

**Table 1:** Frequency of ownership and use of mosquito nets

	Household members			
	N	Own ITN, N (%)	Use ITN, N (%)	^2^ (*P*)
Total	10,891	9182 (84.30)	7217 (66.25)	
Sex				0.492
Boys	2139	1795 (83.92)	1397 (65.31)	
Girls	2092	1772 (84.70)	1402 (67.02)	
Women	6660	5615 (84.31)	4418 (66.34)	
Age group				
Children				
< 1	1162	996 (85.71)	833 (71.69)	
1	784	647 (82.53)	538 (68.62)	
2	830	715 (86.14)	558 (67.23)	
3	689	565 (82.00)	408 (59.22)	
4	766	644 (84.07)	462 (60.31)	
Women				
15–29	3793	3176 (83.73)	2319 (61.14)	
30–39	1800	1549 (86.06)	1346 (74.78)	
40–49	1067	890 (83.41)	753 (70.57)	
Province				< 0.001
Kigali city	1402	1312 (93.58)	1103 (78.67)	
South	2800	2442 (87.21)	1960 (70.00)	
West	2473	1784 (72.14)	1371 (55.44)	
North	1687	1376 (81.56)	1014 (60.11)	
East	2529	2268 (89.68)	1769 (69.95)	
Residence				< 0.001
Urban	2593	2332 (89.93)	1951 (75.24)	
Rural	8298	6850 (82.55)	5266 (63.46)	
Education level				0.001
No education	4873	4027 (82.64)	3188 (65.42)	
Primary	4302	3597 (83.61)	2829 (65.76)	
Secondary & higher	1688	1536 (91.00)	1185 (70.20)	
Missing	28	22 (78.57)	15 (53.57)	
Wealth category				< 0.001
Very low	2330	1606 (68.93)	1204 (51.67)	
Low	2146	1702 (79.31)	1253 (58.39)	
Middle	1980	1712 (86.46)	1340 (67.68)	
Higher	1896	1773 (93.51)	1421 (74.95)	
Highest	2539	2389 (94.09)	1999 (78.73)	
Season				0.0388
Dry	6935	5818 (83.89)	4546 (65.55)	
Rainy	3956	3364 (85.04)	2671 (67.52)	
Altitude				< 0.001
< 1700	6303	5719 (90.73)	4630 (73.46)	
> 1700	4588	3463 (75.48)	2587 (56.39)	

^2^ (*P*): Chi Squared *P* value

Table 2 shows that rural areas had a high proportion of malaria-positive cases (6.51%, *P* > 0.001). The Eastern Province accounted for more than half of all malaria cases in the country and the highest prevalence rate (11.11%), whereas Northern Province and Kigali had the lowest prevalence of malaria in all of the provinces in Rwanda, with only 0.59% and 1.21% respectively. Those with the lowest income were more likely to have malaria, with 8.92% testing positive, whereas individuals with the highest income were the least likely to have malaria (1.45%). People with no education or living at < 1700 m asl also had increased prevalence of malaria with, 7.24 and 7.85%, p < 0.001, respectively.

**Table 2:** Background characteristics of study population according to malaria status

Background characteristics	Study number, N = 10,891	Malaria prevalence rate (%) 5.45	^2^ (*P*)
Sex			< 0.001*
Boy	2139	8.22	
Girl	2092	7.45	
Women	6660	3.93	
Age groups			< 0.001*
< 1	1162	4.99	
1	784	10.20	
2	830	7.46	
3	689	8.56	
4	766	9.53	
14–29	3793	4.56	
30–39	1800	3.0	
40–49	1067	3.28	
Residence			< 0.001*
Urban	2593	2.04	
Rural	8298	6.51	
Province			< 0.001*
Kigali	1402	1.21	
Southern	2800	7.85	
Western	2473	2.66	
Northern	1687	0.59	
Eastern	2529	11.11	
Wealth quintile			< 0.001*
Very low	2330	8.92	
Low	2146	6.80	
Middle	1980	5.90	
Higher	1896	4.53	
Highest	2539	1.45	
Altitude			< 0.001*
< 1700	6303	7.85	
> 1700	4588	2.15	
Educational level			< 0.001*
No education	4873	7.24	
Primary	4302	4.60	
Secondary & more	1688	2.36	
Missing	28		
Slept under mosquito net			< 0.001*
Yes	7217	4.49	
No	3674	6.42	
Season			0.3031
Dry	6935	5.27	
Rainy	3956	5.76	

2 (*P*): Chi Squared *P* value

*Index of statistically significant values for *P* < 0.05

### Logistic regression of factors associated with the prevalence of malaria in Rwanda

Non-use of ITNs was significantly associated with the prevalence of malaria (AOR = 1.33, 95% CI 1.09–1.63), as was living at < 1700 m asl (AOR = 3.59, 95% CI 2.40–4.35). The lowest wealth category was associated with the prevalence of malaria (AOR = 1.50; 95% CI 1.47–1.98). Season and type of residence were not associated with the prevalence of malaria.

## Discussion

There has been a lot of interest about factors driving the persistence of malaria in Rwanda despite various control measures that have been put in place. The determinants of the persistence of malaria in Rwanda were assessed. This study revealed that altitude, non-use of ITNs, and wealth category are associated with malaria, as reported in previous studies in Africa [4, 12, 14].

Non-use of ITNs (AOR = 1.29, 95% CI 1.03–1.60), low altitude (AOR = 2.93, 95% CI 1.94–4.42), and the very low wealth category (AOR 1.54; 95% CI 1.17–2.03) were associated with the prevalence of malaria. Consideration of these factors will facilitate elimination of malaria in Rwanda [15].

**Fig. 1:**
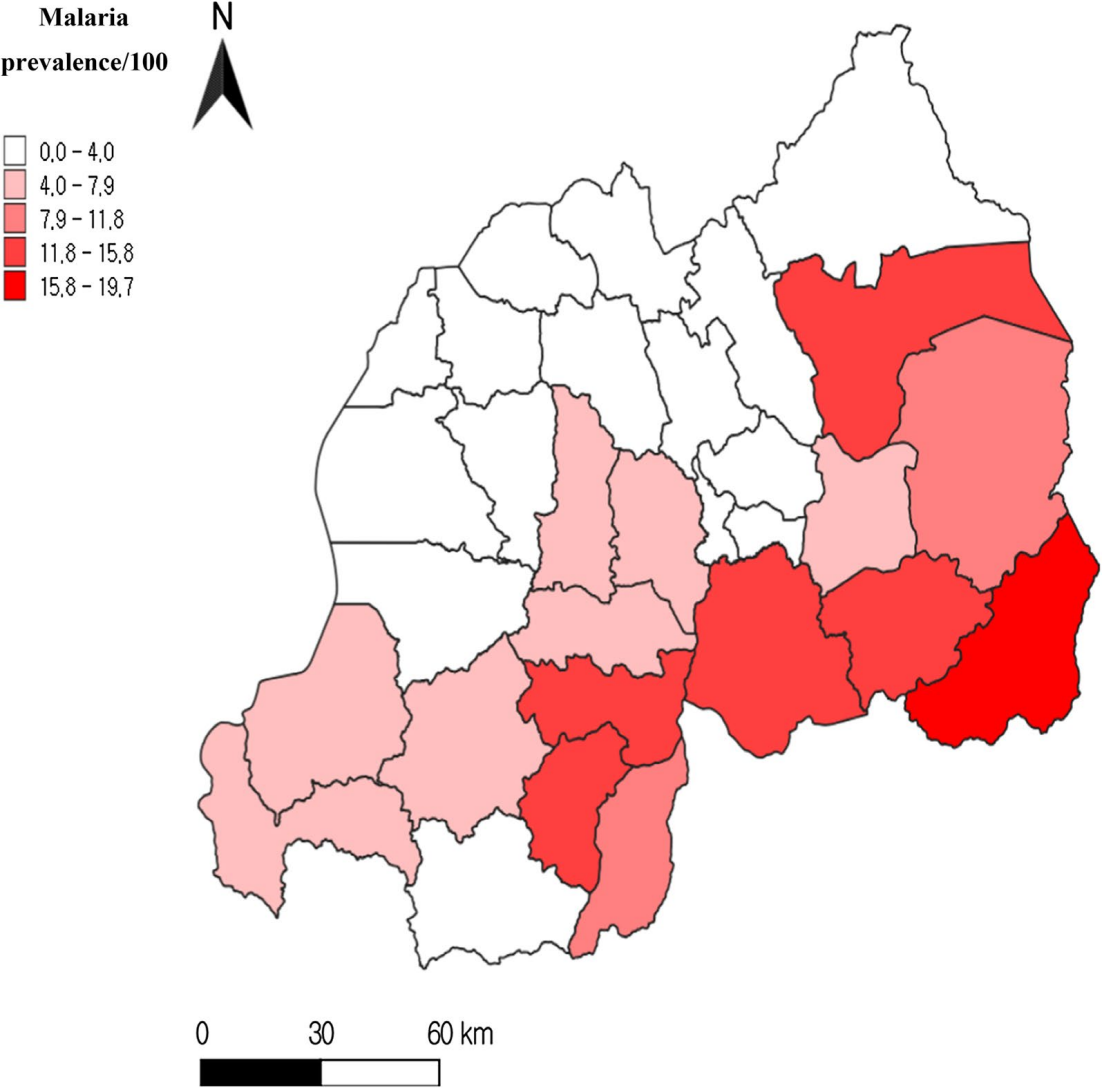
Geographic distribution of malaria cases confirmed by the rapid test. *Only women 15–49 years of age and children < 5 years of age were subjected to rapid malaria testing (10,891)

Environmental parameters play an important role in malaria transmission because they impact the life cycle of the insect vectors and development of the parasite. The probability of malaria transmission decreases with increasing altitude [16]; for example, in Tanzania [12, 17]. In this study, the regions affected by malaria were those at < 1700 m (Fig. 1), particularly the southern and Eastern Provinces (Table 2) Temperature decreases with increasing altitude and a high temperature favours breeding and survival of the insect vectors of malaria. The prevalence of malaria increased with decreasing altitude; people living at < 1700 m asl were at greater prevalence of testing positive to malaria (AOR = 2.93, 95% CI 1.94–4.42) (Table 3).

**Table 3:** Logistic regression of factors associated with the prevalence of malaria

Characteristics	Crude OR (95% CI)	Adjusted OR^a^ (95% CI)
Sex		
Boys	Ref	Ref
Girls	0.92 (0.72–1.16)	0.92 (0.72–1.17)
Women	0.47 (0.37–0.58)	*0.52 (0.41–0.65)*
Age		
< 1	Ref	Ref
1	2.12 (1.49–3.02)	*2.35 (1.63–3.40)*
2	1.45 (1.00–2.11)	*1.47 (1.00–2.16)*
3	1.72 (1.20–2.47)	*1.69 (1.16–2.45)*
4	1.95 (1.36–2.80)	*1.98 (1.38–2.83)*
15–29	0.91 (0.66–1.25)	*0.92 (0.56–1.52)*
30–39	0.56 (0.38–0.84)	*0.56 (0.34–0.92)*
49–49	0.63 (0.40–1.00)	*0.57 (0.34–0.95)*
Use of mosquito nets		
Yes	Ref	Ref
No	1.31 (1.07–1.61)	*1.29 (1.03–1.60)*
Altitude		
< 1700 m	3.92 (2.70–5.69)	*2.93 (1.94–4.42)*
> 1700 m	Ref	Ref
Type of residence		
Rural	Ref	Ref
Urban	0.27 (0.17–0.45)	0.83 (0.46–1.50)
Household wealth quintile		
Very low	1.47 (1.13–1.91)	*1.54 (1.17–2.03)*
Low	1.12 (0.84–1.54)	1.18 (0.89–1.57)
Middle	Ref	Ref
Higher	0.66 (0.48–0.92)	*0.66 (0.48–0.92)*
Highest	0.22 (0.15–0.33)	*0.36 (0.22–0.60)*
Province of residence		
Kigali city	0.43 (0.22–0.87)	0.63 (0.29–1.38)
Southern province	3.12 (1.86–5.25)	*2.84 (1.70–4.83)*
Northern province	0.25 (0.11–0.55)	*0.38 (0.17–0.84)*
Eastern province	4.61 (2.74–7.74)	*3.09 (1.79–5.45)*
Western province	Ref	Ref
Education level		
No education	Ref	Ref
Primary education	0.63 (0.51–0.76)	1.20 (0.82–1.75)
Secondary and higher	0.35 (0.24–0.51)	1.10 (0.62–1.97)
Season		
Dry	Ref	Ref
Rainy	1.11 (0.80–1.54)	1.01 (0.76–1.34)

^**a**^ OR: adjusted for cluster survey design, socioeconomic characteristics, seasonality, altitude, use of mosquito nets, and residence

Italics: statistically significant (*P* < 0.05)

Low income status is linked to the prevalence of malaria [18]. The prevalence of malaria in Uganda reportedly increases with decreasing wealth category [5, 19], and we found that poverty was associated with the prevalence of malaria in Rwanda (AOR = 1.54, 95% CI 1.17–2.03). This may be because the housing of the poverty-stricken is of a standard that facilitates mosquito proliferation. Previous study in Rwanda found out that people living in houses with poor housing wall materials and households affected by famine and droughts are more likely to develop malaria [9]. In addition, low income population mainly live in rural areas where the physical and financial access to health services and facility is not as good as in urban areas. Additionally, the costs of consultation, transportation, and drugs at distant health facilities may be prohibitive for poor families. Poverty can lead to employment in mining or agriculture in the jungle and/or forest, where there is an abundance of malaria vectors. Poverty also stimulates unplanned and rapid migration to undeveloped and densely populated peri-urban areas with poorly constructed housing, which facilitate breeding of mosquitos [19, 20]. Thus, low-quality housing and a lack of knowledge of malaria enable disease transmission among the poverty stricken. Malaria can also be seen as a source of poverty in developing countries, as the majority of the population of such countries depends on agriculture and the rate of transmission of malaria coincides with the planting and harvesting seasons. This can result in economic and nutritional consequences for individual households and the wider community [21].

The use of ITNs is an effective malaria-control strategy, and it is influenced by various factors [22, 23]. Of the population of Rwanda, 84.30% own mosquito nets, but only 66.25% use them (Table 1). The frequency of use of ITNs increased with increasing wealth category (Table 1) as very low income utilize ITNs at a lowest rate compared to very high income population. Non-use of ITNs was associated with an increased prevalence of malaria, in agreement with a prior report that use of ITNs can prevent the transmission of malaria [14].

Residing in Kigali and the Northern Province was negatively associated with testing positive to malaria (AOR = 0.63, 95% CI (0.29–1.38) and AOR = 0.38, 95% CI (0.17–0.84), respectively); however, the prevalence of malaria was high in the Southern and Eastern Provinces (AOR = 3.09, 95% CI (1.79–5.45) and AOR = 2.84, 95% CI (1.70–4.83), respectively). This was due in part to the fact that the eastern and southern parts of Rwanda have the lowest elevation in Rwanda. The Northern Province is the coldest part of the country, which can affect the proliferation of the mosquitoes that spread malaria. Additionally, Kigali is the capital of Rwanda and has more health-related infrastructure than any other place; moreover, its inhabitants are literate, and their income levels are higher than elsewhere, which contribute to decreased vulnerability. Household income is a determinant of where to reside. In developing countries, poor households are typically found in rural areas with no electricity and few health facilities, close to wetlands and forests as they depend on agriculture. Such areas tend to favour mosquito proliferation, leading to a risk of malaria transmission.

Education plays a key role in sustainable response to malaria, and the probability of dying from malaria decreases with increasing education level [4]. This study confirmed the role of education in malaria reduction, as the risk of malaria decreased as the level of education increased. However, in this study, type of residence and season were not associated with the prevalence of malaria. This is because trained community health workers are available in rural areas, which tend to have a high proportion of uneducated people, and provide anti-malarials to symptomatic individuals, particularly children < 5 years of age. There were no significant difference between rainy and dry season which may due to the fact that the mean temperatures were similar in both the rainy and the dry seasons (19.67 °C and 19.63 °C, respectively), whereas the precipitation was higher during the rainy season (132.40 versus 94.77 mm) [24].

## Limitations and strengths

The 2014–2015 Rwanda Demographic Health Survey was of a cross–sectional design, and so causation cannot be inferred; this precludes drawing firm conclusions as to the directions of any associations. In addition, malaria testing was only performed in women and children < 5 years of age rather than in the entire population; this precludes generalization of the associations to the entire population. The survey was performed during 7 months excluding strong dry months; therefore, there were no information on malaria in Rwanda during marked dry season (June–August). Despite these limitations, this study finding provides insight into the prevalence of malaria and the related factors in Rwanda. The effects of non-use of mosquito nets, altitude, and wealth category on the prevalence of malaria has been revealed. This study finding can be used as a reference for future studies and to support the implementation of policies and programmes that aim to eradicate malaria in Rwanda.

## Conclusions

Few studies have assessed the associations of malaria with altitude, season, and non-use of mosquito nets among the general population of Rwanda. This study confirms that altitude, poverty, and non-use of ITNs are associated with malaria prevalence. This reaffirms the importance of taking these factors into account when formulating malaria eradication policies and measures. Longitudinal studies are needed to evaluate the associations of season, and type of residence with the prevalence of malaria, which were non-significant in this study. Future interventions to reduce financial barriers to preventive measures and vulnerability to malaria will reduce the prevalence of malaria. Effective use of ITNs is needed, thus increasing the community resilience on ITNs utilization is needed and areas at low altitudes should be considered when formulating strategies to control and/or eradicate malaria. Finally, the Eastern and Southern Provinces should emphasize malaria-eradication programs, as they are more vulnerable than the other provinces.

## Data Availability

DHS datasets are publicly available on www.dhsprogram.org.

## Abbreviations

RDHS: Rwanda Demographic and Health Survey
ITN: insecticide-treated nets
LLINs: long-lasting insecticidal nets
RBC: Rwanda Biomedical Center
MoH: Ministry of Health
UNICEF: United Nations International Children’s Emergency Fund,
NISR: National Institute of Statistics of Rwanda
NRL: National Reference Laboratory
ICF International: Inner City Fund International
EA: enumeration areas
RPHC: Rwanda Population and Housing Census
